# Exploring the mechanisms of biofield therapy through joint electrophysiological recordings in humans and mice

**DOI:** 10.1016/j.ibneur.2026.05.011

**Published:** 2026-05-28

**Authors:** Arnaud Delorme, Andrew Cusimano, Megan Tran, Phuong Nguyen, Defeng Deng, Chris Fields, Libor Velíšek, Richard Wagner, Peiying Yang, Lorenzo Cohen

**Affiliations:** a The University of Texas MD Anderson Cancer Center, Houston, TX, USA; b Institute of Noetic Sciences, Petaluma, CA, USA; c University of California, La Jolla, San Diego, CA, USA; d Independent Researcher, 11160 Caunes Minervois, France; e New York Medical College, Valhalla, NY, USA

**Keywords:** Biofield Therapy (BT), Mice, Double-blind, Electroencephalogram (EEG)

## Abstract

In this case study, a self-described biofield therapy (BT) therapist and a sham therapist participated in multiple (n = 24) treatment and control (non-treatment) sessions under double-blind conditions. During the treatment phases, BT and sham therapists attempted to influence mice with cancer and control mice, alternating BT with rest phases where no such efforts were made. Both the 64-channel EEG of the human participants and the simultaneous 3-channel EEG and 1-channel EMG of the mice were recorded. For human participants and for the analysis of human and mouse EEG comodulation, the EEG experimental setup was a 2 × 2 design, contrasting mouse type (cancer vs. control) against session type (BT vs. non-treatment; N = 8 in each of the four groups). For the mice EEG, the experimental setup was a 2x2x2 design, contrasting mouse type (cancer vs. control) against session type (treatment vs. non-treatment) and human participant (BT participant vs. sham participant). Although no changes in spectral power were detected in mice, a significant increase in theta band coherence indicates that this type of biofield therapy may influence large-scale neural coordination rather than localized activity. Concurrently, robust and reproducible alterations in the therapist’s EEG across all frequency bands during treatment periods, irrespective of mouse condition, suggest a consistent physiological signature associated with the act of intentional BT. Treatment was associated with changes in EEG coherence and spectral correlation between human and mouse signals. In particular, we observed an interaction in which treatment differentially affected brain-to-brain coherence in cancer versus control mice. These findings describe condition-dependent alterations in coupled physiological measures and suggest a complex relationship between human and mouse neural activity during BT sessions, while remaining agnostic about the underlying mechanism. We also outline the study's limitations and potential for follow-up investigations, acknowledging that these exploratory physiological findings do not have any clinical implications and should not be interpreted as justification for cancer treatment or as a substitute for evidence-based medical care.

## Background

Once considered a marginal field, certain complementary and alternative medicine (CAM) interventions have demonstrated therapeutic benefits significant enough to be integrated into mainstream medical practice. However, skepticism persists for some therapies due to limited scientific understanding of their biological mechanisms. Biofield therapy (BT), categorized as an “energy therapy” by the National Cancer Institute’s Office of Complementary and Integrative Health, is one such approach (Categories of CAM Therapies et al., n.d.). Practitioners of BT claim to influence health and biological functions through non-contact methods such as Reiki, Healing Touch, Therapeutic Touch, and Qigong (Baime, 2000, Ebneter et al., 2001, Güthlin et al., 2012, Jain and Hammerschlag, 2015). Although the mechanisms underlying BT remain unclear, it is widely practiced, and many individuals believe in its efficacy, with patients often reporting positive experiences (Baime, 2000, Ebneter et al., 2001, Güthlin et al., 2012).

Research evaluating BT has produced mixed results. Some clinical trials suggest that therapies like Therapeutic Touch, Healing Touch, and Reiki contribute to improvements in subjective measures such as pain relief and anxiety reduction, as well as potential immunological benefits (Jain et al., 2015). However, the overall body of evidence remains inconclusive. A systematic review by Rao et al. (2016) identified 27 studies on BT for symptom management in non-communicable diseases, with 13 reporting significant outcomes. In contrast, other reviews argue that most findings support the idea that BT’s effects do not extend beyond placebo responses (Ernst, 2003). A meta-analysis by Astin et al. (2000) found that approximately 57% of studies reported positive treatment outcomes, yet methodological flaws such as small sample sizes, uncontrolled baseline differences, patient variability, and reliance on subjective endpoints weakened their validity. The overall study quality, measured by the Jadad score, averaged 3.6 out of 5, suggesting that many trials fell short of rigorous scientific standards (Astin et al., 2000), although a more recent meta-analysis provided a more favorable assessment (Roe et al., 2015). Focused reviews in oncology and palliative or end-of-life care similarly indicate that biofield therapies may provide symptom relief in selected contexts, yet there was a high degree of heterogeneity in study design, outcome measures, and statistical rigor (Henneghan and Schnyer, 2015, Tabatabaee et al., 2016). Systematic evaluation of mental health applications suggests that effects above placebo may exist for certain outcomes, but that conclusions remain tentative due to limited high quality randomized trials and variable risk of bias (Zadro and Stapleton, 2022). A comprehensive scoping review mapping the clinical research landscape across biofield modalities documented the breadth of published studies while identifying persistent gaps in methodology, reporting consistency, and mechanistic understanding, as well as cultural and institutional barriers that complicate integration into mainstream biomedical frameworks (Sprengel et al., 2025). In parallel, newly proposed reporting guidelines tailored to biofield trials argue that improved transparency, intervention specification, and protocol standardization are necessary to enhance reproducibility and comparability across studies, particularly given variability in practitioner training and delivery (Hammerschlag et al., 2024).

Despite these challenges, more methodologically rigorous investigations have sought to establish a clearer picture of BT’s effectiveness. Jain et al. (2012), for example, conducted a double-blind study on 76 breast cancer survivors and observed significant biomarker changes in those receiving BT compared to a placebo. Lutgendorf et al. (2010) similarly found that Therapeutic Touch produced measurable differences in both patient-reported experiences and immune cell activity. More recently, we investigated the effects of biofield therapy (BT) on pancreatic cancer cells and the physiological responses of a BT practitioner, using a double-blind, controlled design with 60 treatment sessions to assess changes in cell activity and practitioner EEG and HRV. The study revealed significant physiological shifts, correlations between EEG and cellular markers, and a potential causal relationship between the practitioner’s neural activity and cellular changes (Cohen et al., 2024). These findings suggest that BT may have measurable physiological effects, particularly when studies control for potential biases by eliminating physical contact between the practitioner and recipient.

To improve the reliability of BT research, studies should focus on isolating specific treatment modalities, utilizing animal models or biological tissue rather than human subjects to minimize placebo effects, and addressing practitioner variability. Because BT practices can differ greatly among individuals, case studies may provide valuable insights by focusing on detailed empirical observations rather than averaging effects across heterogeneous populations. Similar to our previous study that targeted pancreatic cancer cells (Cohen et al., 2024), the present study applies this approach to an animal model, aiming to detect subtle effects that larger trials might miss.

In animal models, past studies found BT suppressed tumor metastasis and modified immune responses (Gronowicz et al., 2015). In studies involving mouse models of Lewis lung carcinoma, BT appeared to slow tumor progression and increase apoptosis and necrosis (Yang et al., 2019, Yang et al., 2020). Additional findings suggested that BT could alter the tumor microenvironment and impact cancer cell stemness (Yang et al., 2020). More recent preliminary work identified significant effects on cancer cell proliferation, motility, invasiveness, membrane potential, and protein expression (Yang et al., 2022, Yang et al., 2024).

Despite this growing body of research, no widely accepted scientific framework explains how BT exerts its effects. Several theoretical models related to non-reductionist consciousness models have been proposed to align with BT’s principles (Kuhn, 2024). Establishing a comprehensive theoretical basis will likely require extensive experimentation to identify commonalities across different studies. Rather than attempting to confirm a specific mechanism, the present study aimed to determine whether BT produces measurable effects in both control and cancer mice with spontaneous liver tumors in laboratory settings.

Prior studies suggest that shifts in a BT practitioner’s physiological state might correspond to therapeutic effects (Baime, 2000, Baldwin and Hammerschlag, 2014, Güthlin et al., 2012, Matos et al., 2021). To explore this possibility, we monitored brain activity through electroencephalography (EEG), aiming to determine whether measurable physiological shifts aligned with therapeutic intent. EEG in mice and BT practitioner (or sham practitioner) was collected simultaneously and synchronized to millisecond precision. This approach is based on the premise that mental states and autonomic regulation, as reflected by EEG patterns, may have an impact on biological processes.

This study involved a single BT practitioner conducting blinded treatment sessions on live mice with cancer, with control conditions including live mice without cancer. Data on sham-treated control mice (with and without cancer) was also collected to account for potential placebo-like effects. The primary objectives were to assess the impact of BT on mouse brain activity, examine physiological changes in the practitioner during treatment, and investigate possible correlations between these factors.

Our study tested three key pre-registered hypotheses (Effect of biofield therapy on the EEG in a mouse model of cancer, 2022): 1) whether the EEG of mice changes during BT compared to sham treatment, whether baseline EEG differences exist between cancerous and non-cancerous mice, and whether these differences persist during the therapy; 2) whether the BT practitioner’s EEG undergoes changes during treatment and whether these changes vary over time depending on whether the treated mice have cancer or belong to the control group; 3) whether there was an association between the EEG activity of the BT practitioner (or sham practitioner) and the treated mice during BT. Specifically, Hypothesis 3 examined whether the EEG patterns of the practitioner and the mice were time-synchronized in real-time during treatment and whether this synchronization differed depending on whether the treated mice had cancer or belonged to the cancer-free control group.

## Methods

The protocol was pre-registered with Open Science Framework https://osf.io/n2gwj/wiki/home/. The present manuscript reports findings from the electrophysiological recordings. Outcomes related to data collection of blood, tumour tissue, and brain tissue will be reported in a separate publication as relevant. The human subjects portion of the study was approved by the Institutional Review Board of The University of Texas MD Anderson Cancer Center (protocol 2020–1210; Start and end dates of the study recruitment were 11/28/2022–12/03/2022). The BT participant and control participant provided written informed consent before any data collection. All data collection of mouse EEG data was conducted at Baylor College of Medicine as part of a service agreement under the leadership of Dr. Jianrong Tang, Baylor College of Medicine, with animal use protocol AN−5585.

### Participant

The first participant was an adult BT therapist using the Bengston Energy Healing Method, and this was the only biofield technique employed in the study; all reported results pertain exclusively to this method. Central to the Bengston method is the practice of Image Cycling, a process that involves rapidly cycling through a series of mental images of personal desires or outcomes (Bengston and Krinsley, 2000, Bengston and Moga, 2007). This practice is designed to enhance the treatment process by engaging the practitioner's focus and energy in a dynamic and fast-paced manner. The technique is mechanical and devoid of any specific belief system. Also, the treatment intent is not a formed or focused concentration on the target, as the practitioner claims he tries to “get out of the way.” The BT participant was a 70-year-old male at the time of the study and had over 13 years of experience in the Bengston method and over 40 years of experience with other modalities. In addition to his years of practice with human recipients, the BT practitioner had prior training experience performing healing interventions with non-human subjects, including mice, which is now explicitly noted to contextualize his familiarity with this experimental setting. The second participant acted as a sham therapist to control for the presence of a person and to collect EEG data and examine changes over time. The sham participant was a 52-year-old male who had no experience in any BT modality. They were provided instructions on the distance to sit from the mice and movements to make in order to mimic the movements of the BT participant. No instructions were provided on the use of any mental imagery.

### Procedure

**Fig. 1:**
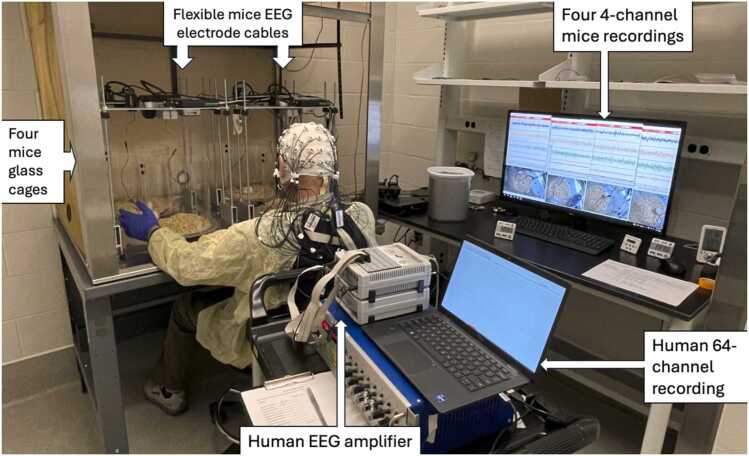
Experimental setup. BT participant provides treatment to either control or cancer mice, four at a time. Both human EEG and mice EEG are recorded simultaneously. The human EEG amplifier and mice EEG amplifiers are synchronized to millisecond precision using TTL pulses (see Methods).

Twenty-four sessions were provided by the BT participant and the sham control participant (12 each). Each session produces two EEG files. The first file is a baseline of 10 min when the mice are not present yet (these files are not synchronized). A second period starts with a baseline of 3 min, followed by a treatment period of about 30 min, and then a 10-minute post-baseline period starts after the end of the treatment period. The session is shown in Fig. 1 and Fig. 3. The red segment of the graph represents the baseline and is saved as a separate file. During the baseline, the participants are in a different room, and the EEG recordings are not synchronized between humans and mice. All the blue segments (pre-baseline, treatment, post-baseline) are contained as a second independent file synchronized between humans and mice (see below). During the pre- and post-baseline of the blue segment, the mice were present, but the human participant turned their back on them.

A total of 24 sessions are acquired over 6 days, conducting 4 sessions per day. Based on the diagram of Fig. 3, a total of 48 files were collected, corresponding to 96 different periods. Half of the sessions had mice with cancer, and half of the sessions contained control mice. The experimenters interacting with the participant, participants, and data analysts were blind to the type of mice.

### Human physiology data collection

Human EEG data were collected using an ActiChamp Plus 64 System (BrainProduct Inc.) with a sampling rate of 500 Hz. The 64-channel actiCAP Snap cap was used with electrode names following the 10–20 nomenclature (Fig. 1). During cap preparation, electrode impedances were kept below 25 kOhms following the BrainProduct company recommendations.

### Mice physiology data collection

A total of 32 mice were included in the study, consisting of 16 LPA-tTA/Tet-O-MYC transgenic mice that developed spontaneous liver tumors and 16 wildtype littermate control mice without cancer, which were randomized to the BT or sham conditions according to the preregistered design (8 mice in each of four groups). Baseline body weights were comparable between groups at study initiation (Supplementary Table 7). Activity levels during the experimental sessions were not measured and are not reported here. All the transgenic mice developed liver tumour nodules, as confirmed by gross examination of excised liver tissues after the 10 day study and great liver weights in the transgenic relative to the wild type, with no differences between BT and sham. There were also no group differences in body weights at the end of the study. Activity levels during the sessions were not examined. No medications or conventional cancer treatments were administered to mice in either group.

**Fig. 2:**
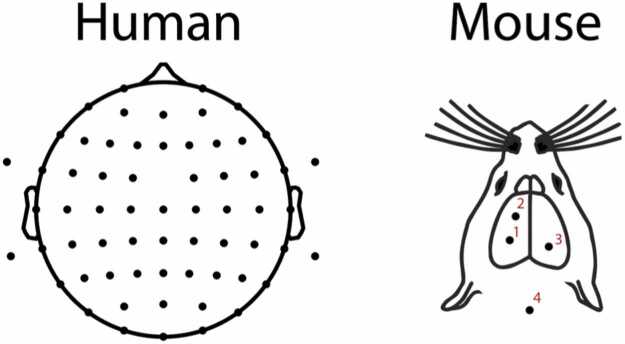
Electrode configurations for human and mouse participants are depicted. Human subjects were instrumented with a 64-channel actiCAP system adhering to the 10–10 electrode placement convention. Mouse subjects underwent implantation of four electrodes: Channel 1 (CH1) was positioned in the subdural space of the left parietal cortex, Channel 2 (CH2) in the subdural space of the left frontal cortex, Channel 3 (CH3) in the right CA1 hippocampus, and Channel 4 (CH4) in the neck musculature for electromyographic (EMG) recording (refer to the Methods section for detailed procedures). Note that in Fig. 5, Fig. 6, electrodes located below the midline and thus outside the head outlines have been omitted for visual clarity.

Each mouse was implanted with the following electrodes under aseptic conditions for a total of 4 channels of field potential recordings: CH1 (left parietal cortex), Teflon-coated silver wire (bare diameter 127 µm, A-M systems) was implanted in the subdural space of the left parietal cortex (Paxinos and Franklin, 2019) (0.8 mm posterior, 1.8 mm lateral to the bregma, reference at the midline over the cerebellum); CH2 (left frontal cortex), Teflon-coated silver wire (bare diameter 127 µm, A-M systems) was implanted in the subdural space of the left frontal cortex (Paxinos and Franklin, 2019) (1.8 mm anterior, 1.5 mm lateral to the bregma, reference at the midline over the cerebellum); CH3 (right CA1), Teflon-coated tungsten wire (bare diameter 50 µm, A-M systems) was stereotaxically implanted in the CA1 of the hippocampus (Paxinos and Franklin, 2019) (2.00 mm posterior, 1.20 mm lateral, and 1.35 mm below the bregma, reference in the ipsilateral corpus callosum); CH4 (neck muscles), Teflon-coated silver wires (bare diameter 127 µm, A-M systems) were implanted in the neck muscles for the recording of electromyography (reference also implanted in the neck muscle). All the electrode wires, together with the attached miniature connector sockets, were fixed on the skull by dental cement, as shown in Fig. 2. After 2–3 weeks of post-surgical recovery, each mouse received one BT session every other day for a total of 3 sessions using the Sirenia data acquisition system (Pinnacle Tech). Signals were amplified (10x), filtered (bandpass, 0.5 Hz – 2.5 kHz), and digitized at 5 kHz. While video-EEG recordings were also acquired, they were not included in this particular analysis.

**Fig. 3:**
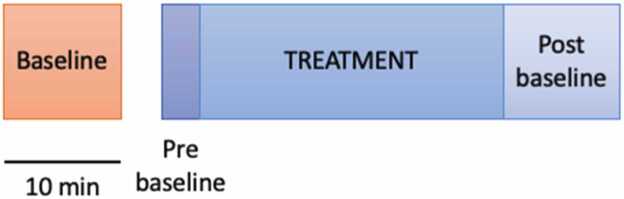
Experimental procedure. A BT session was recorded in two files: a 10-minute baseline file (red segment) where mice are absent and the therapist/sham participant is in another room, and a second file (blue segments) containing a 3-minute pre-baseline, a roughly 30-minute treatment period (with participant-defined end), and a 10-minute post-baseline.

### Animal model

We used LPA-tTA/Tet-O-MYC transgenic mice in this study, described previously (Shachaf et al., 2004). TRE-MYC (Tet-O-MYC)/FVB mice were crossed with LAP-tTA/mice, backcrossed with FVB mice for more than 10 generations. When male LPA-tTA/Tet-O-MYC mice were 8 weeks old, doxycycline was removed from the drinking water. The TRE-MYC mice (no cancer) were used as controls. After a 2-week recovery from EEG implantation, the spontaneous liver cancers in LPA-tTA/Tet-O-MYC transgenic mice were formed based on our experience and published study (Shachaf et al., 2004), and biofield therapy began. Groups of mice were subjected to three treatments each. The statistical model accounted for this repeated treatment regime.

### Data marking and synchronization

Manual annotations for each phase of the experiment were added to the recording traces for experimental treatments for both human and mouse EEG using a Master−8 event trigger box (A.M.P.I.). Experimenter AC inserted event markers in the data file during recording for later data segmentation (beginning of pre-baseline, beginning of treatment, beginning of post-baseline). The box sent millisecond-precision TTL pulses simultaneously to both the human EEG and mouse EEG amplifiers, with multiple events for each recording. Custom cabling was developed to interface the TTL ports of the human and mouse EEG amplifiers; because synchronization relied on standard 5 V TTL pulses referenced to ground, the cabling was electrically simple and served primarily to ensure connector compatibility and reliable signal transmission. These events were used to synchronize the human and mouse EEG down to millisecond precision (see below). Note that the baseline files for mice and humans are recorded in different rooms and have no common markers (red periods in Fig. 3), so they cannot be synchronized to millisecond precision.

**Fig. 4:**
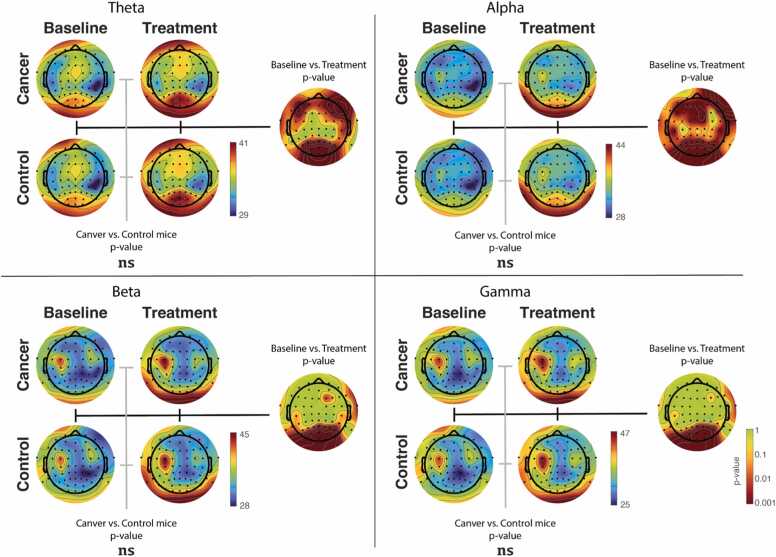
BT participant EEG spectral power in all frequency bands in the 2 × 2 ANOVA design (mouse type x treatment conditions). Spectral power is shown in the central region of each panel, and significance is shown on the right for the treatment effect and below for the mouse type effect after correction for multiple comparisons. The significance scale (after FDR correction for multiple comparisons) is shown using a logarithmic scale. Here, Baseline refers to the average of the 3 baseline periods shown in Fig. 3.

### EEG data preprocessing

Experimenters AC and AD imported the EEG BrainVision files and segmented the different data epochs. Segment extraction was performed based on the manual markers. Experimenters AC and AD were blind to the type of mice (cancer vs. no cancer) presented to the BT and sham control participants. The data segments were double-checked for length and position using automated scripts, ensuring millisecond precision between human and mouse EEG recordings. We then high-pass filtered the data at 3 Hz using a Butterworth 4th-order filter (transition band from 2.75 Hz to 3.25 Hz; 3 Hz was selected to avoid potential low-frequency artifacts because the lowest frequency being studied is theta from 4 to 8 Hz). The *clean*_*rawdata* EEGLAB (Delorme and Makeig, 2004) plugin (v2.7) was used to detect bad channels with correlation thresholds of 0.8 and bad segments of data with thresholds of 20 standard deviations. This plugin uses the Artifact Subspace Reconstruction (ASR) method to detect and correct bad portions of data. We only used ASR’s algorithm as a detection method and removed the bad data segments instead of correcting them (the default in EEGLAB for offline processing). Specifically for human data (but not joint mice and human data processed together), we applied Independent Component Analysis (Picard algorithm, standard approach) and the *ICLabel* EEGLAB plugin (v1.4) to detect bad components. *ICLabel* is a machine-learning algorithm that detects artifactual ICA components based on their topography and activity. Each component is assigned a probability of belonging to 1 of 7 classes, which include the muscle and eye movement artifact classes. We applied the *ICLabel* default method to detect eye and muscle artifacts with probability thresholds of 0.9 (on average, one or two components were rejected for each dataset). This type of pipeline is optimal for maximizing significance in EEG experiments (Delorme, 2023).

Spectral processing was performed using EEGLAB's *std_spec* function using default FFT mode (*specmode* option set to *fft*, and *logtrials* option set to *off*). One-second contiguous and non-overlapping windows are extracted and tapered by a Hamming window before computing the FFT. We considered four frequency bands: theta (4–8 Hz), alpha (8–12 Hz), beta (18–22 Hz), and gamma (30–45 Hz). Spectral power is averaged across segments for each frequency, log-transformed, and then averaged again for all frequencies within the selected frequency range.

### Statistical analysis

The experiment was pre-registered on Open Science Framework prior to data collection (https://osf.io/n2gwj/wiki/home/). A total of 32 mice were included in the preregistered design, comprising two primary biological groups of interest: cancer (n = 16) and non-cancer controls (n = 16). Random allocation to treatment and sham conditions created four balanced experimental groups (n = 8 each), but the primary analyses modeled cancer status and treatment as fixed effects rather than treating the four groups as independent. Sample size considerations were based on detecting large standardized differences in EEG outcomes, as small effects would be harder to interpret. For a two-sided α = 0.05 comparison, approximately 16 subjects per biological group provides about 85% power to detect an effect of roughly 1.1 standard deviation units (Cohen’s d ≈ 1.1). For a more moderate effect (d ≈ 0.8), power with this sample size is substantially lower, on the order of ∼60%, indicating that the study is primarily sensitive to large effects.

**Fig. 5:**
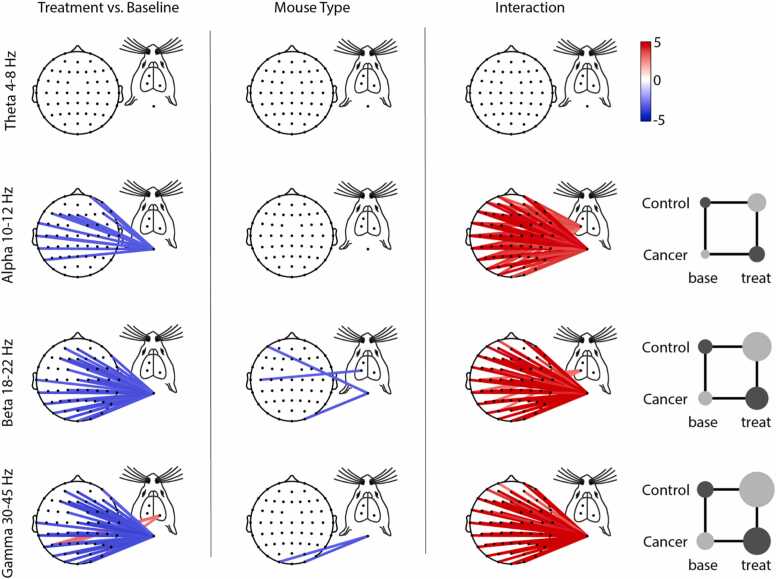
Spectral correlation between mouse and BT participant EEG signals across frequency bands. Each row represents a different frequency band, and columns show the effects of treatment vs. baseline (left), mouse type (middle), and their interaction (right). Columns indicate variables or interaction terms. Lines indicate significant correlations between EEG electrodes in mice and human, color-coded by direction (red: positive; blue: negative). Statistical significance was determined using mixed models and FDR-corrected (see Methods). In the interaction column, marginal means for the significant channel pairs are shown in the adjacent schematic, where node size reflects relative amplitude and color reflects sign (lighter: positive; darker: negative) – lines between dots indicate significance below 0.05. For example, a line between the top left dot (baseline control) and the top right dot (treatment control) indicates a significant difference between these conditions for the electrode pairs that showed a significant interaction effect. Here, Baseline refers to the average of the 3 baseline periods shown in Fig. 3.

**Fig. 6:**
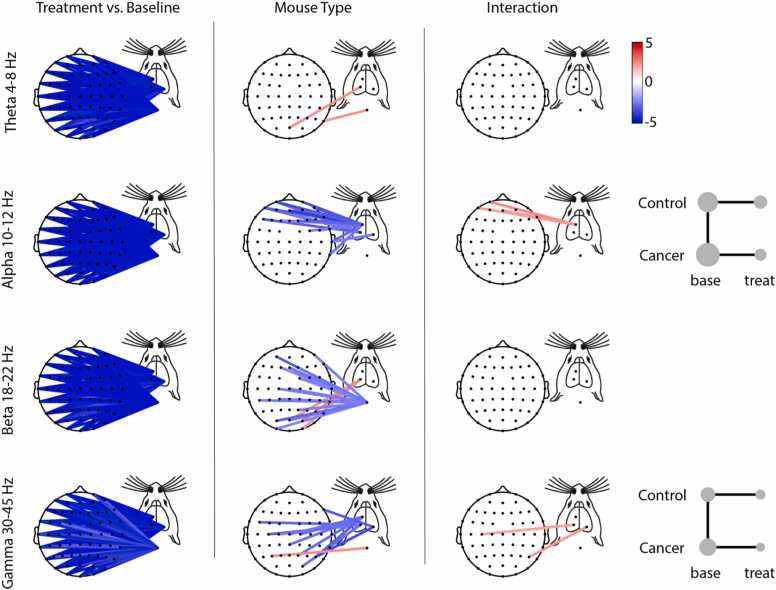
Spectral coherence indicates the synchronization between the mouse and BT participant EEG signals across frequency bands. Each row represents a different frequency band, and columns show the effects of treatment vs. baseline (left), mouse type (middle), and their interaction (right). Lines indicate significant coherence between EEG electrodes in mice and humans, color-coded by direction (red: positive, blue: negative). In the interaction column, marginal means for the significant channel pairs are shown as detailed in Fig. 5. Statistical significance was determined using mixed models and FDR-corrected (see Methods). Here, Baseline refers to the average of the pre- and post-baseline periods shown in Fig. 3.

The statistical analysis addressed three primary questions using linear mixed-effects models implemented with MATLAB’s fitlme function. This framework accounts for within-animal correlation through random effects while estimating fixed effects of cancer status and treatment, improving efficiency relative to single-summary analyses and supporting valid inference in repeated-measures designs with modest sample sizes. First, we examined whether mouse EEG activity was modulated by biofield therapy by testing for differences between treatment and sham conditions, treatment versus baseline periods, and across cancer versus non-cancer mice. Second, we assessed changes in the therapist’s EEG spectral power during treatment compared to baseline, and whether these changes varied depending on the type of mice (cancer or control) being treated. Third, we evaluated the degree of time-synchronized EEG activity between the therapist and mice during treatment by analyzing spectral correlation and coherence, and whether this synchronization differed by mouse group. For all models, fixed effects included experimental condition factors (e.g., treatment, mouse type), while random intercepts accounted for individual variability (e.g., mouse ID). Coherence was computed as the cross-spectrum summed across trials and normalized by the square root of the product of the autospectra, following the EEGLAB implementation (Delorme and Makeig, 2004; Equation 5). Band-level values were obtained by averaging complex coherency across frequencies within each band. The MATLAB code is available at https://github.com/arnodelorme/simple_coherence. To address the issue of multiple comparisons across frequency bands and electrode pairs, we applied the False Discovery Rate (FDR) correction method (Benjamini and Hochberg, 1995). Statistical significance was evaluated using FDR-adjusted p-values with α = 0.05 unless otherwise stated. We used the EEGLAB software FDR function (fdr.m), and we also report the adjusted p-values after multiple-comparison correction. For Fig. 5, Fig. 6, and Supplementary Figures 2 and 3, marginal means and significance were calculated for all electrode pairs in the interaction term at each frequency. When multiple electrode pairs were significant, effects were averaged, and significance was aggregated using Simes’ method (Simes, 1986).

## Results

Based on our pre-registration document (Cohen, 2022), we tested the following questions: (1) Treatment and mouse EEG: Does the EEG of the mouse change during BT versus sham therapy, treatment vs. baseline periods, and cancer vs. non-cancer mice; (2) Treatment and human EEG: Does the EEG of the biofield therapist change during treatment and are there any time effects for the changes in human EEGs when different groups of mice (cancer vs control) are treated; and (3) Mouse EEG and human EEG: Are the mouse and therapist time-synchronized EEGs correlated during biofield therapy in real time, and does this synchronization depend on the groups of mice (cancer vs control)?

Question 1 Treatment and mouse EEG.

Four frequency bands were analyzed: theta (4–8 Hz), alpha (8–12 Hz), narrow band beta range (18–22 Hz), and gamma (30–45 Hz). While these ranges are not ideal for rodents, which have distinct EEG band definitions, we adopted the human conventions to enable joint analysis across species. Our decision was further supported by the fact that key frequency bands, such as theta, which is dominant in rodents and associated with cognitive engagement, have recognized functional analogies across species, similar to human alpha. We used linear mixed-effects models (LME) to examine the effects of experimental variables such as baseline vs. treatment, therapist vs. sham presence, and mouse type (cancer vs. no cancer), as well as their interactions (see Methods). In mouse EEG power, there was no main effect for any of the independent variables or their interactions (Supplementary Table 1). We also observed no effect of spectral power correlation across pairs of channels (Supplementary Table 2). However, we did observe an effect of coherence between channels in the theta frequency band for the baseline vs. treatment condition (Supplementary Table 3). All pairs of electrodes were coherent in all frequency bands at p < 0.002 after correction for multiple comparisons. This effect was not associated with a specific mouse type (cancer vs. control) or participant type (BT vs. sham).

Question 2 Treatment and human EEG.

Changes in EEG spectral power were evaluated during the treatment versus the baseline condition and when the BT participant (or sham participant) was treating cancer and non-cancer mice. This was conducted using a 2 × 2 factorial design (treatment vs baseline) by mouse type (see Fig. 3 and Methods).

In Table 1, the comparison between treatment and baseline showed significant differences across all frequency bands, revealing variations in spectral power during treatment compared to baseline. Significant distinctions were noted in the spectral power of all frequency bands in the occipital regions as a function of treatment. There were no significant main effects of mouse type and no interaction effects, indicating that the influence of mouse type on spectral power did not vary between treatment and baseline.

**Table 1:** BT participants’ EEG spectral power results for treatment x mouse type. The first value in each cell is the maximum statistical value (F) across all channels. The value in parentheses shows the minimum p-value across channels after correction for multiple comparisons (see Methods). Here, Baseline refers to the average of the 3 baseline periods shown in Fig. 3.

**Freq. range**	**Baseline vs. Treat.**	**Cancer vs. Control**	**Interaction**
**Theta 4–8 Hz**	130.83 (p < 0.01)	5.13 (ns)	1.48 (ns)
**Alpha 8 −12 Hz**	179.99 (p < 0.01	6.57 (ns)	1.44 (ns)
**Beta 18–22 Hz**	86.90 (p < 0.01)	6.07 (ns)	0.60 (ns)
**Gamma 30–45 Hz**	65.33 (p < 0.01)	6.07 (ns)	0.79 (ns)

An alternative approach to understanding these findings involves examining EEG scalp power topographies. Fig. 4 illustrates the results presented in Table 1. The main effects of mouse type and the interaction effects are excluded from the figure due to the absence of significant electrodes for the interaction between the two factors across all frequency bands. Supplementary Figure 1 and Supplementary Table 4 show the spectral result for the sham participant, revealing fewer and smaller differences, respectively.

Question 3 Synchronized mouse and human EEG.

Hypothesis 3 tested whether mouse and BT or sham participants’ EEG activity were correlated in real-time during the treatment period, examining both spectral power correlations and spectral coherence, and whether this synchronization differed between cancer and control mouse groups.

The spectral correlation analysis in Fig. 5 revealed significant frequency-specific effects, corrected for multiple comparisons using the False Discovery Rate (FDR). Supplementary Table 4 summarizes the significant electrode pairs. Contrary to the expectation that treatment might enhance cross-species EEG alignment due to shared neurophysiological responses, treatment-induced widespread decreases in spectral correlation in the alpha (10–12 Hz), beta (18–22 Hz), and gamma (30–45 Hz) bands were noted. This reduction suggests a divergence in frequency-specific dynamics between human and mouse EEG signals during treatment, rather than the anticipated convergence. A few significant main effects of mouse type (cancer vs control) were observed after FDR correction in the beta and gamma frequency bands. However, a robust interaction effect was found across all frequency bands, where the pattern of mouse-human EEG correlations differed by mouse type during treatment, with strong positive correlations (red lines) emerging predominantly in the interaction contrasts. The same effects were not noted for the sham participant (Supplementary Table 5 and Supplementary Figure 2).

Spectral coherence analysis in Fig. 6 revealed a significant decrease in mouse-human EEG coherence during treatment compared to baseline across all frequency bands (theta, alpha, beta, gamma), with widespread negative effects observed over the human scalp. Supplementary Table 6 summarizes the significant electrode pairs. These reductions, corrected using FDR, indicate a generalized suppression of interspecies coherence during biofield therapy. In contrast, the main effect of mouse type showed more localized and frequency-specific differences, including a few positive (red) and negative (blue) connections, primarily in the alpha, beta, and gamma bands. Interaction effects were minimal, with only isolated significant connections, suggesting limited modulation of coherence by mouse group during treatment.

Supplementary Tables 5 and 6 summarize the statistical results for the BT participant and the sham participant. Supplementary Figures 2 and 3 show spectral correlation and spectral coherence between the sham participant’s EEG and mice, provided for reference. While quantitative comparison is limited by the small sample of human subjects, a qualitative assessment is possible. Spectral correlation changes between treatment and baseline (Fig. 5) move in opposite directions in the beta and gamma bands for the sham versus BT participant, and more correlations appear specific to the BT participant. For spectral coherence (Fig. 6), the treatment vs. baseline effect is also present with the sham participant (see Discussion). Effects related to mouse type and interaction appear in the theta band, and extend to the alpha, beta, and gamma bands for only the BT participant.

## Discussion

**Fig. 7:**
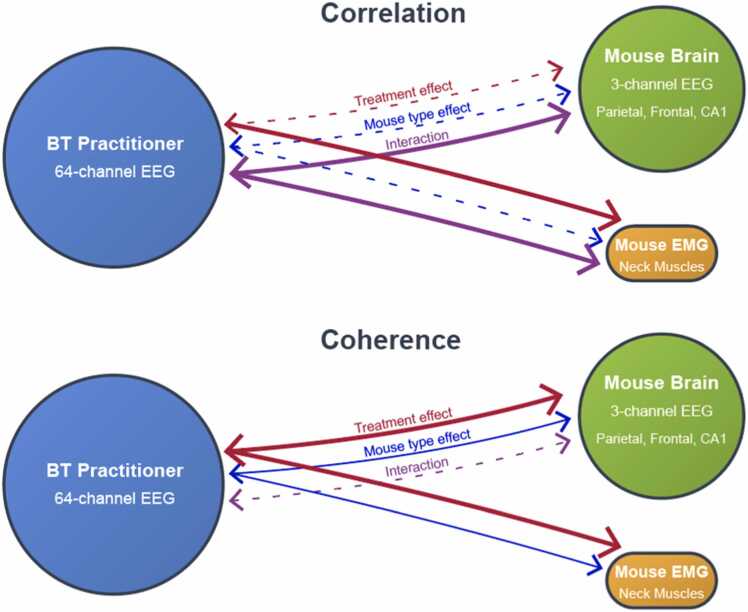
Effect size representations for treatment, mouse type, and their interaction are shown for correlation (Fig. 5) and coherence (Fig. 6). No line indicates no connections. Thin dashed lines indicate 1–4 connections across all frequency bands, thin solid lines 4–100 connections, and thick lines more than 100 connections. A marked pattern difference emerges: the correlation plot shows more connections between human brain and mouse muscle electrodes, whereas the coherence plots highlight more connections for the interaction between human and mouse brain electrodes.

The primary goal of this study was to explore the potential impact of one expert BT practitioner using a rigorous experimental design and advanced analytical techniques. The experimental setup was designed to minimize biases and ensure the accuracy of results. The findings of this study suggest that there may be a complex interplay of physiological responses between humans and mice specific to BT sessions that is not present during sham sessions. The human EEG spectral power showed significant changes during the treatment phase compared to baseline across all frequency bands, indicating an altered physiological state in the biofield therapist and sham, with more and larger targeted changes in the BT participant (Fig. 7). The mouse EEG data did not reveal significant changes in spectral power between treatment and baseline, treatment type, mouse type, or any interactions. Most effects were not apparent during sham treatment. Additionally, the analysis of mouse-human EEG correlations and coherence provided insights into potential interspecies synchronization during BT that were not present during the sham sessions. Mouse EEG and muscle activity both appear correlated with human brain activity, but the most informative are the brain-to-brain connections, as these are less likely to reflect direct interaction or spurious correlations arising from the shared environment or from human presence influencing mouse activity and muscle responses (Fig. 7). There is a noticeable difference, as illustrated in Fig. 7: the correlation plot of Fig. 5 reveals more connections between human brain and mouse muscle electrodes, while the coherence plots of Fig. 6 emphasize increased connections driven by the interaction between human and mouse brain electrodes. This last pattern could reflect an interaction in which treatment selectively alters brain-to-brain coherence in the cancer mice. This result is encouraging and warrants further studies.

### Treatment and mouse EEG

For Question 1, analysis of mouse EEG responses to biofield therapy (BT) revealed no significant changes in spectral power across the four frequency bands (theta, alpha, beta, gamma) when comparing treatment to baseline or sham sessions, nor any interactions involving cancer versus control mice. In rodents, theta is a dominant rhythm—functionally analogous to alpha in humans—and is associated with increased cognitive engagement, primarily mediated by hippocampal generators. Although we applied human EEG frequency definitions to mouse data to enable cross-species comparison, the resulting measures remain interpretable. Our decision was further supported by the fact that key frequency bands, such as theta, have recognized functional analogies across species; for instance, rodent theta is a dominant rhythm often linked to cognitive engagement, similar to human alpha. Although this approach may miss narrow, rodent-specific power changes, our goal was to examine broader patterns of activity and synchrony. Future studies focusing solely on mice could instead employ frequency bands optimized for rodent neurophysiology.

### Treatment and human EEG

The results for Question 2 revealed significant changes in the BT participant’s EEG spectral power during treatment compared to baseline across all frequency bands, suggesting an altered physiological state associated with the therapeutic process. Similar increases in both low- and high-frequency EEG activity have been reported during meditation (Guo et al., 2022). Weaker and less targeted changes were apparent in the sham participant. Notably, these effects were independent of whether the mice were cancerous or not, indicating that the therapist’s neural state was modulated by the act of engaging in the treatment process rather than by the specific condition of the target. This pattern mirrors our previous findings with the same BT participant (Cohen et al., 2024), where similar increases in EEG power were observed during BT and were correlated with shifts in cancer cell activity, suggesting a potential link between neural activation in the therapist and biological outcomes in the target. Both studies reinforce the idea that BT elicits consistent neurophysiological changes in the practitioner that may underlie the therapeutic mechanism, highlighting the reproducibility of EEG markers of treatment intent across different experimental setups and also relative to a sham therapist (Guo et al., 2022).

### Synchronized mouse and human EEG

Analysis of within-mouse EEG coherence revealed a significant increase in theta band coherence during treatment relative to baseline. In contrast, cross-species (mouse–human) coherence, shown in Fig. 6, exhibited a significant decrease during treatment across frequency bands. This could, for example, suggest that while absolute EEG power remained stable, the presence of a person may influence the coordination or synchrony of neural activity within the mouse brain. For example, increased theta coherence in humans has been associated with altered states of consciousness and integrative neural processing (Clayton et al., 2018). The absence of effects tied to mouse cancer status or therapist condition underscores that this coherence shift might be a general physiological response to the presence of a person in a therapeutic context rather than a targeted effect. These results are consistent with prior research suggesting that non-contact human presence can influence biological systems through mechanisms not reflected in standard spectral power measures (Baldwin & Hammerschlag, 2014; Gronowicz, Bengston, et al., 2015), emphasizing the need to examine neural synchrony metrics in such studies.

Marginal main effects of mouse type were observed for spectral correlation. Interaction effects indicated that the pattern of EEG connectivity varied depending on the treatment group, either cancer or control mice. This implies that the degree and nature of synchronization may depend on the physiological state of the target, aligning with theories suggesting that BT effects are mediated through subtle, dynamic informational exchanges rather than direct energy transfer. Importantly, these same interaction effects were not found for the sham participant.

Alternatively, the coherence observed in Fig. 6 for the treatment factor can be interpreted as a state of stable, spontaneous coupling between the human and mouse EEG signals prior to the onset of biofield therapy. This coherence may reflect low-frequency physiological alignment or shared environmental entrainment when no active treatment occurs during baseline. During BT and sham, the observed decrease in coherence and spectral correlation suggests a disruption or reorganization of this baseline synchrony, potentially due to a shift in each participant’s attentional or cognitive state, which could modulate interspecies neural dynamics. This pattern is consistent with prior research showing that intentional mental states can alter the coupling between individuals’ EEGs (Grinberg-Zylberbaum et al., 1994) and that cognitive effort or altered consciousness can reduce low-frequency synchrony while enhancing more focused, high-frequency dynamics (Tallon-Baudry and Bertrand, 1999). A noteworthy observation is that spectral correlation between human EEG and mouse electromyography (EMG) exhibited alterations during the course of treatment, but also based on the type of mice. Specifically, these changes were observed in the cancer cohort compared to the control group, as depicted in the second column of Fig. 5. Although such changes could reflect altered motor mobility in cancer-afflicted mice, an alternative explanation is a specific treatment effect on mouse behavior, supported by the significant brain-to-brain connections in the third interaction column. The latter is more likely, as there were few to no such associations between mouse type EEG and sham participant EEG. These findings are promising and call for further investigation.

These findings build on earlier observations of correlated physiological activity across therapist-recipient pairs (Baldwin and Hammerschlag, 2014) and theoretical models proposing consciousness-based mechanisms for BT (Fields et al., 2024). Moreover, the modulation of cross-brain coherence during BT echoes prior reports of time-locked neural synchronization between spatially separated systems under focused intention (Grinberg-Zylberbaum et al., 1994). Together, these results contribute to a growing body of evidence that challenge conventional biological paradigms and warranting further investigation using advanced neurophysiological tools.

### Limitations

The present study has important limitations. It investigated only one BT modality and a single experienced practitioner compared with one sham control, which substantially limits the ability to generalize the results. While the repeated-measures design across multiple mice allowed for within-subject comparisons, these results should be interpreted as exploratory and hypothesis-generating rather than definitive. Findings cannot be generalized to all BT modalities or practitioners, as inter-practitioner variability is likely substantial, and practitioner-specific factors might influence the observed outcomes. Because the study relied on a single BT practitioner, any physiological changes measured may reflect idiosyncratic traits, techniques, or neural responses unique to that individual rather than general properties of BT. Future studies should include multiple practitioners across different BT modalities to assess whether the patterns observed here are consistent or practitioner-dependent.

While we observed significant treatment-related changes in coherence and cross-species correlations, the interpretation of these findings requires caution. Both BT and sham conditions were associated with widespread alterations relative to baseline, indicating that some of the observed synchrony changes may reflect nonspecific factors such as the mere presence of a human, or shared sensory cues rather than effects unique to BT. Therefore, although the reduction in cross-species coherence during BT compared to baseline was robust, we cannot conclusively attribute this to a specific biofield mechanism. Electromagnetic effects observed in animals and humans should also be considered (Wang et al., 2019, Nordmann et al., 2026). An alternative explanation is that cancer-related physiological or behavioral differences in the mice, together with changes in therapist attentional or cognitive state during active treatment, could independently contribute to the observed alterations in cross-species EEG coherence, underscoring that these findings do not uniquely imply a treatment-specific mechanism. Future studies should include additional control conditions and improved blinding to disentangle nonspecific influences from effects that may be unique to BT.

Additionally, the false discovery rate method used to correct for multiple comparisons showed sensitivity to the extent of significance. Specifically, the widespread coherence between BT and sham participants and mouse EEG in the treatment versus baseline condition (Fig. 6) affected the significance threshold for other comparisons, such as mouse type and interaction effects. To our knowledge, there is no established correction method to account for this issue, and it remains an observation rather than a demonstrated fact. This highlights the need for further statistical research to identify appropriate correction methods for this type of analysis.

## Conclusion

In conclusion, this study provides novel insights into the physiological dynamics of a given BT modality by capturing simultaneous EEG recordings from a practitioner and live animal recipients under controlled, double-blind, repeated conditions. While no changes in spectral power were observed in mice, increased theta band coherence suggests that the specific BT modality may modulate functional connectivity between human and mouse brains. Significant shifts in the therapist’s EEG during treatment, independent of mouse condition, point to a repeatable physiological state associated with therapeutic intent that was not seen in the sham participant. Moreover, changes in mouse-human EEG coherence specific for cancer mice during treatment suggest a dynamic reorganization of interspecies synchrony. These findings build upon prior evidence and highlight the need for further investigation into the underlying mechanisms of BT using rigorous electrophysiological and statistical methodologies. We acknowledge that these exploratory physiological findings do not have any clinical implications and should not be interpreted as justification for cancer treatment or as a substitute for evidence-based medical care. Future work should build on this paradigm through replication, the inclusion of diverse BT methods, multiple practitioners, and refined analytical frameworks to better characterize and interpret subtle interspecies bioelectrical interactions.

## CRediT authorship contribution statement

**Megan Tran:** Data curation. **Andrew Cusimano:** Data curation. **Defeng Deng:** Data curation. **Phuong Nguyen:** Data curation. **Lorenzo Cohen:** Writing – review & editing, Funding acquisition, Conceptualization. **Peiying Yang:** Writing – review & editing, Data curation, Conceptualization. **Arnaud Delorme:** Writing – review & editing, Writing – original draft, Formal analysis, Data curation, Conceptualization. **Richard Wagner:** Data curation. **Velisek Libor:** Formal analysis, Data curation. **Chris Fields:** Writing – review & editing, Conceptualization.

## Ethics declarations

The protocol was pre-registered with Open Science Framework https://osf.io/n2gwj/wiki/home/. The human aspects of the study were approved by the Institutional Review Board of The University of Texas MD Anderson Cancer Center (protocol code 2020–1210). The BT and control participants provided informed consent before any data collection. The mouse EEG procedures and data collection was conducted under animal use protocol AN−5585, Baylor College of Medicine.

## Funding

This research was funded by the Emerald Gate Charitable Trust, grant number RCTS LS2022−00061373-LK.

## Declaration of Competing Interest

The authors declare that they have no known competing financial interests or personal relationships that could have appeared to influence the work reported in this paper.

## Data Availability

The data was converted to the BIDS EEG format (Pernet et al., 2018) using the *EEG-BIDS* plugin of the EEGLAB software (Delorme and Makeig, 2004). It is large (350 Gb) and available upon request.

## Footnotes

Appendix A Supplementary data associated with this article can be found in the online version at doi:10.1016/j.ibneur.2026.05.011.
