# Supplementary material for: Exploring the mechanisms of biofield therapy through joint electrophysiological recordings in humans and mice
Source: IBRO Neurosci Rep. 2026 May 28;21:52–62. doi: 10.1016/j.ibneur.2026.05.011 (PMC13266234; doi:10.1016/j.ibneur.2026.05.011)
Supplement: Supplementary file 1 — Supplementary material [file mmc1.docx]

|  |  | **Chan 1** | **Chan 2** | **Chan 3** | **Chan 4 (EMG)** |
| --- | --- | --- | --- | --- | --- |
| **baseline vs. treatment (Treatment)** | **theta** | 0.0000 (ns) | 0.0000 (ns) | 0.0000 (ns) | 0.0000 (ns) |
|  | **alpha** | 0.0000 (ns) | 0.0000 (ns) | 0.0000 (ns) | 0.0000 (ns) |
|  | **beta** | 0.0000 (ns) | 0.0000 (ns) | 0.0000 (ns) | 0.0000 (ns) |
|  | **gamma** | 0.0000 (ns) | 0.0001 (ns) | 0.0001 (ns) | 0.0001 (ns) |
| **therapist vs. sham (Therapist)** | **theta** | 0.0000 (ns) | 0.0001 (ns) | 0.0004 (ns) | 0.0087 (ns) |
|  | **alpha** | 0.0001 (ns) | 0.0001 (ns) | 0.0001 (ns) | 0.0088 (ns) |
|  | **beta** | 0.0001 (ns) | 0.0000 (ns) | 0.0001 (ns) | 0.0105 (ns) |
|  | **gamma** | 0.0000 (ns) | 0.0000 (ns) | 0.0001 (ns) | 0.0121 (ns) |
| **cancer vs. control (Cancer)** | **theta** | 0.0005 (ns) | 0.0000 (ns) | 0.0011 (ns) | 0.0000 (ns) |
|  | **alpha** | 0.0006 (ns) | 0.0001 (ns) | 0.0006 (ns) | 0.0000 (ns) |
|  | **beta** | 0.0018 (ns) | 0.0007 (ns) | 0.0014 (ns) | 0.0000 (ns) |
|  | **gamma** | 0.0011 (ns) | 0.0011 (ns) | 0.0018 (ns) | 0.0001 (ns) |
| **Treatment x Therapist** | **theta** | 0.0000 (ns) | 0.0000 (ns) | 0.0000 (ns) | 0.0000 (ns) |
|  | **alpha** | 0.0000 (ns) | 0.0000 (ns) | 0.0000 (ns) | 0.0000 (ns) |
|  | **beta** | 0.0000 (ns) | 0.0000 (ns) | 0.0000 (ns) | 0.0000 (ns) |
|  | **gamma** | 0.0000 (ns) | 0.0000 (ns) | 0.0000 (ns) | 0.0000 (ns) |
| **Cancer x Therapist** | **theta** | 0.0000 (ns) | 0.0000 (ns) | 0.0000 (ns) | 0.0000 (ns) |
|  | **alpha** | 0.0000 (ns) | 0.0000 (ns) | 0.0000 (ns) | 0.0000 (ns) |
|  | **beta** | 0.0000 (ns) | 0.0000 (ns) | 0.0000 (ns) | 0.0000 (ns) |
|  | **gamma** | 0.0000 (ns) | 0.0000 (ns) | 0.0000 (ns) | 0.0001 (ns) |
| **Cancer x Treatment** | **theta** | 0.0139 (ns) | 0.0131 (ns) | 0.0070 (ns) | 0.0306 (ns) |
|  | **alpha** | 0.0174 (ns) | 0.0130 (ns) | 0.0049 (ns) | 0.0279 (ns) |
|  | **beta** | 0.0208 (ns) | 0.0164 (ns) | 0.0088 (ns) | 0.0301 (ns) |
|  | **gamma** | 0.0201 (ns) | 0.0191 (ns) | 0.0105 (ns) | 0.0306 (ns) |
| **Cancer x Treatment x Therapist** | **theta** | 0.0040 (ns) | 0.0038 (ns) | 0.0036 (ns) | 0.0039 (ns) |
|  | **alpha** | 0.0040 (ns) | 0.0037 (ns) | 0.0037 (ns) | 0.0039 (ns) |
|  | **beta** | 0.0040 (ns) | 0.0039 (ns) | 0.0038 (ns) | 0.0036 (ns) |
|  | **gamma** | 0.0041 (ns) | 0.0040 (ns) | 0.0039 (ns) | 0.0038 (ns) |

***Supplementary Table 1.*** *Mice's power spectrum across frequency bands (theta, alpha, beta, gamma) for each experimental condition. Effect sizes are reported, with corrected p-values shown in parentheses. Significant differences after multiple comparison correction are indicated by exact p-values; non-significant results are marked as (ns).*

|  |  | **C1 x C2** | **C1 x C3** | **C1 x C4** | **C2 x C3** | **C2 x C4** | **C3 x C4** |
| --- | --- | --- | --- | --- | --- | --- | --- |
| **baseline vs. treatment (Treatment)** | **theta** | 0.0000 (ns) | 0.0000 (ns) | 0.0000 (ns) | 0.0000 (ns) | 0.0000 (ns) | 0.0000 (ns) |
|  | **alpha** | 0.0000 (ns) | 0.0000 (ns) | 0.0000 (ns) | 0.0000 (ns) | 0.0001 (ns) | 0.0000 (ns) |
|  | **beta** | 0.0001 (ns) | 0.0000 (ns) | 0.0001 (ns) | 0.0000 (ns) | 0.0001 (ns) | 0.0000 (ns) |
|  | **gamma** | 0.0004 (ns) | 0.0001 (ns) | 0.0000 (ns) | 0.0001 (ns) | 0.0000 (ns) | 0.0000 (ns) |
| **therapist vs. sham (Therapist)** | **theta** | 0.0000 (ns) | 0.0000 (ns) | 0.0000 (ns) | 0.0000 (ns) | 0.0000 (ns) | 0.0003 (ns) |
|  | **alpha** | 0.0000 (ns) | 0.0000 (ns) | 0.0000 (ns) | 0.0000 (ns) | 0.0000 (ns) | 0.0002 (ns) |
|  | **beta** | 0.0000 (ns) | 0.0000 (ns) | 0.0000 (ns) | 0.0000 (ns) | 0.0000 (ns) | 0.0000 (ns) |
|  | **gamma** | 0.0003 (ns) | 0.0000 (ns) | 0.0001 (ns) | 0.0000 (ns) | 0.0000 (ns) | 0.0000 (ns) |
| **cancer vs. control (Cancer)** | **theta** | 0.0000 (ns) | 0.0001 (ns) | 0.0001 (ns) | 0.0000 (ns) | 0.0002 (ns) | 0.0000 (ns) |
|  | **alpha** | 0.0001 (ns) | 0.0001 (ns) | 0.0002 (ns) | 0.0000 (ns) | 0.0003 (ns) | 0.0000 (ns) |
|  | **beta** | 0.0000 (ns) | 0.0000 (ns) | 0.0001 (ns) | 0.0000 (ns) | 0.0002 (ns) | 0.0000 (ns) |
|  | **gamma** | 0.0001 (ns) | 0.0000 (ns) | 0.0000 (ns) | 0.0001 (ns) | 0.0000 (ns) | 0.0000 (ns) |
| **Treatment x Therapist** | **theta** | 0.0000 (ns) | 0.0000 (ns) | 0.0000 (ns) | 0.0000 (ns) | 0.0000 (ns) | 0.0000 (ns) |
|  | **alpha** | 0.0000 (ns) | 0.0000 (ns) | 0.0000 (ns) | 0.0000 (ns) | 0.0000 (ns) | 0.0000 (ns) |
|  | **beta** | 0.0000 (ns) | 0.0000 (ns) | 0.0000 (ns) | 0.0000 (ns) | 0.0001 (ns) | 0.0000 (ns) |
|  | **gamma** | 0.0001 (ns) | 0.0000 (ns) | 0.0001 (ns) | 0.0000 (ns) | 0.0001 (ns) | 0.0001 (ns) |
| **Cancer x Therapist** | **theta** | 0.0000 (ns) | 0.0000 (ns) | 0.0000 (ns) | 0.0000 (ns) | 0.0000 (ns) | 0.0000 (ns) |
|  | **alpha** | 0.0000 (ns) | 0.0000 (ns) | 0.0000 (ns) | 0.0000 (ns) | 0.0000 (ns) | 0.0000 (ns) |
|  | **beta** | 0.0000 (ns) | 0.0000 (ns) | 0.0000 (ns) | 0.0000 (ns) | 0.0001 (ns) | 0.0000 (ns) |
|  | **gamma** | 0.0002 (ns) | 0.0000 (ns) | 0.0000 (ns) | 0.0000 (ns) | 0.0000 (ns) | 0.0000 (ns) |
| **Cancer x Treatment** | **theta** | 0.0017 (ns) | 0.0005 (ns) | 0.0000 (ns) | 0.0003 (ns) | 0.0003 (ns) | 0.0007 (ns) |
|  | **alpha** | 0.0025 (ns) | 0.0003 (ns) | 0.0003 (ns) | 0.0001 (ns) | 0.0003 (ns) | 0.0004 (ns) |
|  | **beta** | 0.0012 (ns) | 0.0005 (ns) | 0.0000 (ns) | 0.0001 (ns) | 0.0001 (ns) | 0.0008 (ns) |
|  | **gamma** | 0.0006 (ns) | 0.0009 (ns) | 0.0000 (ns) | 0.0004 (ns) | 0.0000 (ns) | 0.0013 (ns) |
| **Cancer x Treatment x Therapist** | **theta** | 0.0005 (ns) | 0.0002 (ns) | 0.0001 (ns) | 0.0001 (ns) | 0.0001 (ns) | 0.0003 (ns) |
|  | **alpha** | 0.0005 (ns) | 0.0002 (ns) | 0.0001 (ns) | 0.0001 (ns) | 0.0001 (ns) | 0.0003 (ns) |
|  | **beta** | 0.0002 (ns) | 0.0002 (ns) | 0.0000 (ns) | 0.0001 (ns) | 0.0000 (ns) | 0.0004 (ns) |
|  | **gamma** | 0.0000 (ns) | 0.0004 (ns) | 0.0002 (ns) | 0.0002 (ns) | 0.0000 (ns) | 0.0006 (ns) |

***Supplementary Table 2.*** *Pairwise intra-mice comparisons of cross-spectrum across frequency bands (theta, alpha, beta, gamma) for each experimental condition. Columns indicate channel pairs (C1 is for channel 1, etc.). Effect sizes are reported, with corrected p-values shown in parentheses. Significant differences after multiple comparison correction are indicated by exact p-values; non-significant results are marked as (ns).*

|  |  | **C1 x C2** | **C1 x C3** | **C1 x C4** | **C2 x C3** | **C2 x C4** | **C3 x C4** |
| --- | --- | --- | --- | --- | --- | --- | --- |
| **baseline vs. treatment (Treatment)** | **theta** | 0.0291 (0.003) | 0.0446 (0.0001) | 0.0459 (9e-5) | 0.0380 (0.0004) | 0.0493 (6e-5) | 0.0602 (1e-5) |
|  | **alpha** | 0.0571 (2e-5) | 0.0540 (3e-5) | 0.0531 (4e-5) | 0.0397 (0.0003) | 0.0341 (0.0009) | 0.0394 (0.0003) |
|  | **beta** | 0.0318 (0.001) | 0.0494 (6e-5) | 0.0659 (9e-6) | 0.0305 (0.002) | 0.0673 (9e-6) | 0.0574 (2e-5) |
|  | **gamma** | 0.0526 (4e-5) | 0.0437 (0.0001) | 0.0552 (3e-5) | 0.0353 (0.0007) | 0.0304 (0.002) | 0.0496 (6e-5) |
| **therapist vs. sham (Therapist)** | **theta** | 0.0026 (ns) | 0.0090 (ns) | 0.0015 (ns) | 0.0030 (ns) | 0.0027 (ns) | 0.0023 (ns) |
|  | **alpha** | 0.0036 (ns) | 0.0001 (ns) | 0.0007 (ns) | 0.0034 (ns) | 0.0061 (ns) | 0.0055 (ns) |
|  | **beta** | 0.0008 (ns) | 0.0007 (ns) | 0.0063 (ns) | 0.0005 (ns) | 0.0014 (ns) | 0.0051 (ns) |
|  | **gamma** | 0.0000 (ns) | 0.0000 (ns) | 0.0014 (ns) | 0.0013 (ns) | 0.0062 (ns) | 0.0018 (ns) |
| **cancer vs. control (Cancer)** | **theta** | 0.0003 (ns) | 0.0000 (ns) | 0.0000 (ns) | 0.0016 (ns) | 0.0007 (ns) | 0.0020 (ns) |
|  | **alpha** | 0.0016 (ns) | 0.0005 (ns) | 0.0005 (ns) | 0.0107 (ns) | 0.0044 (ns) | 0.0008 (ns) |
|  | **beta** | 0.0015 (ns) | 0.0001 (ns) | 0.0000 (ns) | 0.0039 (ns) | 0.0006 (ns) | 0.0002 (ns) |
|  | **gamma** | 0.0012 (ns) | 0.0002 (ns) | 0.0064 (ns) | 0.0006 (ns) | 0.0012 (ns) | 0.0008 (ns) |
| **Treatment x Therapist** | **theta** | 0.0020 (ns) | 0.0016 (ns) | 0.0012 (ns) | 0.0021 (ns) | 0.0012 (ns) | 0.0012 (ns) |
|  | **alpha** | 0.0000 (ns) | 0.0000 (ns) | 0.0001 (ns) | 0.0007 (ns) | 0.0022 (ns) | 0.0026 (ns) |
|  | **beta** | 0.0027 (ns) | 0.0004 (ns) | 0.0004 (ns) | 0.0013 (ns) | 0.0001 (ns) | 0.0021 (ns) |
|  | **gamma** | 0.0010 (ns) | 0.0001 (ns) | 0.0000 (ns) | 0.0007 (ns) | 0.0027 (ns) | 0.0000 (ns) |
| **Cancer x Therapist** | **theta** | 0.0003 (ns) | 0.0002 (ns) | 0.0010 (ns) | 0.0024 (ns) | 0.0000 (ns) | 0.0006 (ns) |
|  | **alpha** | 0.0002 (ns) | 0.0005 (ns) | 0.0004 (ns) | 0.0033 (ns) | 0.0010 (ns) | 0.0007 (ns) |
|  | **beta** | 0.0001 (ns) | 0.0002 (ns) | 0.0003 (ns) | 0.0008 (ns) | 0.0005 (ns) | 0.0003 (ns) |
|  | **gamma** | 0.0000 (ns) | 0.0005 (ns) | 0.0006 (ns) | 0.0009 (ns) | 0.0000 (ns) | 0.0000 (ns) |
| **Cancer x Treatment** | **theta** | 0.0029 (ns) | 0.0041 (ns) | 0.0027 (ns) | 0.0017 (ns) | 0.0097 (ns) | 0.0000 (ns) |
|  | **alpha** | 0.0015 (ns) | 0.0000 (ns) | 0.0030 (ns) | 0.0023 (ns) | 0.0029 (ns) | 0.0019 (ns) |
|  | **beta** | 0.0009 (ns) | 0.0014 (ns) | 0.0038 (ns) | 0.0004 (ns) | 0.0021 (ns) | 0.0092 (ns) |
|  | **gamma** | 0.0008 (ns) | 0.0007 (ns) | 0.0002 (ns) | 0.0000 (ns) | 0.0000 (ns) | 0.0017 (ns) |
| **Cancer x Treatment x Therapist** | **theta** | 0.0019 (ns) | 0.0005 (ns) | 0.0015 (ns) | 0.0030 (ns) | 0.0056 (ns) | 0.0001 (ns) |
|  | **alpha** | 0.0001 (ns) | 0.0001 (ns) | 0.0003 (ns) | 0.0012 (ns) | 0.0014 (ns) | 0.0021 (ns) |
|  | **beta** | 0.0004 (ns) | 0.0002 (ns) | 0.0002 (ns) | 0.0000 (ns) | 0.0002 (ns) | 0.0020 (ns) |
|  | **gamma** | 0.0000 (ns) | 0.0003 (ns) | 0.0000 (ns) | 0.0005 (ns) | 0.0003 (ns) | 0.0000 (ns) |

***Supplementary Table 3.*** *Pairwise intra-mice comparisons of mice coherence across frequency bands (theta, alpha, beta, gamma) and channel pairs (C1 is for channel 1, etc.) for each experimental condition. Effect sizes are reported, with corrected p-values shown in parentheses. Significant differences after multiple comparison correction are indicated by exact p-values; non-significant results are marked as (ns). The “baseline vs. treatment” condition shows widespread significant increases across all bands, while other contrasts (e.g., therapist vs. sham, cancer vs. control, and interaction effects) yield mostly non-significant results.*

| **Freq. range** | **Baseline vs. Treat.** | **Cancer vs. Control** | **Interaction** |
| --- | --- | --- | --- |
| **Theta 4 - 8 Hz** | 69.78 (p<0.01) | 2.58 (ns) | 0.35 (ns) |
| **Alpha 8 -12 Hz** | 43.19 (p<0.01) | 1.14 (ns) | 0.38 (ns) |
| **Beta 18 - 22 Hz** | 70.49 (p<0.01) | 4.83 (ns) | 1.05 (ns) |
| **Gamma 30 - 45 Hz** | 93.36 (p<0.01) | 5.17 (ns) | 1.13 (ns) |

***Supplementary Table 4.*** *Sham participants’ EEG spectral power results for treatment x mouse type. The first value in each cell is the maximum statistical value (F) across all channels. The value in parentheses shows the minimum p-value across channels after correction for multiple comparisons (see Methods).*

|  |  |  | **Chan 1** | **Chan 2** | **Chan 3** | **Chan 4 (EMG)** |
| --- | --- | --- | --- | --- | --- | --- |
| **BT participant** | **baseline vs. treatment (Treatment)** | **theta** | -0.02 (0%) | 0.70 (0%) | 0.48 (0%) | 0.30 (0%) |
|  |  | **alpha** | -2.30 (0%) | -0.00 (0%) | 1.45 (0%) | -2.82 (22%) |
|  |  | **beta** | -1.06 (0%) | 0.36 (0%) | 1.22 (0%) | -3.01 (60%) |
|  |  | **gamma** | 0.46 (0%) | 1.74 (0%) | 2.27 (6%) | -3.20 (70%) |
|  | **cancer vs. control (Cancer)** | **theta** | -1.33 (0%) | 1.03 (0%) | 0.54 (0%) | -0.58 (0%) |
|  |  | **alpha** | -2.42 (0%) | -0.90 (0%) | 0.25 (0%) | -2.14 (0%) |
|  |  | **beta** | -1.91 (2%) | -1.24 (0%) | -0.14 (0%) | -2.36 (3%) |
|  |  | **gamma** | -0.77 (0%) | -0.61 (0%) | 0.34 (0%) | -2.47 (3%) |
|  | **Interaction** | **theta** | 0.79 (0%) | -0.23 (0%) | -0.24 (0%) | 0.70 (0%) |
|  |  | **alpha** | 3.83 (98%) | 0.80 (0%) | -1.08 (0%) | 4.76 (100%) |
|  |  | **beta** | 2.61 (10%) | 0.84 (0%) | -0.44 (0%) | 5.18 (98%) |
|  |  | **gamma** | 1.10 (0%) | -0.30 (0%) | -1.04 (0%) | 5.48 (100%) |
| **Sham Participant** | **baseline vs. treatment (Treatment)** | **theta** | 0.13 (0%) | 0.10 (0%) | 0.26 (0%) | 0.18 (0%) |
|  |  | **alpha** | 0.49 (2%) | 0.34 (0%) | 0.61 (2%) | 0.09 (0%) |
|  |  | **beta** | 2.33 (29%) | 1.10 (0%) | 1.80 (3%) | 0.73 (0%) |
|  |  | **gamma** | 2.92 (38%) | 1.65 (13%) | 1.93 (11%) | 0.94 (0%) |
|  | **cancer vs. control (Cancer)** | **theta** | -0.51 (0%) | -0.75 (0%) | -0.73 (0%) | -0.41 (0%) |
|  |  | **alpha** | -0.77 (0%) | -0.91 (0%) | -0.67 (0%) | -0.81 (0%) |
|  |  | **beta** | 0.08 (0%) | -0.59 (0%) | 0.57 (0%) | -0.34 (0%) |
|  |  | **gamma** | -0.07 (0%) | -0.89 (0%) | 0.51 (0%) | -0.62 (0%) |
|  | **Interaction** | **theta** | 0.47 (0%) | 0.53 (0%) | 0.35 (0%) | 0.38 (0%) |
|  |  | **alpha** | 0.28 (0%) | 0.47 (0%) | 0.04 (0%) | 0.62 (0%) |
|  |  | **beta** | -1.01 (3%) | 0.28 (0%) | -1.26 (0%) | 0.38 (0%) |
|  |  | **gamma** | -1.28 (14%) | 0.29 (0%) | -1.26 (0%) | 0.34 (0%) |

***Supplementary Table 5.*** *Pairwise comparisons of 64-channel human EEG spectral power and mice spectral power at 4 electrode sites across frequency bands (theta, alpha, beta, gamma) for each experimental condition. Average effect sizes are reported, with the percentage of significant (after multiple comparison correction) pairwise comparisons in parentheses.*

|  |  |  | **Chan 1** | **Chan 2** | **Chan 3** | **Chan 4 (EMG)** |
| --- | --- | --- | --- | --- | --- | --- |
| **BT participant** | **baseline vs. treatment (Treatment)** | **theta** | -4.20 (100%) | -5.19 (100%) | -4.97 (100%) | -5.45 (100%) |
|  |  | **alpha** | -6.15 (100%) | -5.96 (100%) | -5.80 (100%) | -5.87 (100%) |
|  |  | **beta** | -5.10 (100%) | -5.08 (100%) | -4.95 (100%) | -6.02 (100%) |
|  |  | **gamma** | -5.93 (100%) | -5.58 (100%) | -5.19 (100%) | -4.16 (100%) |
|  | **cancer vs. control (Cancer)** | **theta** | 0.12 (2%) | -0.45 (0%) | -0.40 (0%) | 0.12 (2%) |
|  |  | **alpha** | -0.94 (6%) | -1.39 (25%) | -0.77 (2%) | -0.47 (0%) |
|  |  | **beta** | -0.06 (5%) | -0.41 (0%) | -0.15 (2%) | -1.41 (22%) |
|  |  | **gamma** | -0.95 (8%) | -0.83 (8%) | -0.49 (3%) | 0.41 (2%) |
|  | **Interaction** | **theta** | -0.07 (0%) | 0.27 (0%) | 0.32 (0%) | -0.01 (0%) |
|  |  | **alpha** | 0.74 (0%) | 1.07 (6%) | 0.73 (0%) | 0.48 (0%) |
|  |  | **beta** | -0.18 (0%) | 0.13 (0%) | 0.21 (0%) | 0.90 (0%) |
|  |  | **gamma** | 0.65 (2%) | 0.60 (0%) | 0.24 (2%) | -0.42 (0%) |
| **Sham Participant** | **baseline vs. treatment (Treatment)** | **theta** | -4.98 (100%) | -5.00 (100%) | -4.58 (100%) | -4.76 (100%) |
|  |  | **alpha** | -4.70 (100%) | -4.69 (100%) | -4.16 (100%) | -4.54 (100%) |
|  |  | **beta** | -4.32 (100%) | -4.38 (100%) | -4.16 (100%) | -4.33 (100%) |
|  |  | **gamma** | -4.00 (100%) | -4.22 (100%) | -4.80 (100%) | -4.46 (100%) |
|  | **cancer vs. control (Cancer)** | **theta** | 0.34 (5%) | -0.76 (3%) | -1.01 (17%) | 0.13 (2%) |
|  |  | **alpha** | -0.56 (0%) | -0.77 (2%) | 0.39 (0%) | -0.02 (0%) |
|  |  | **beta** | 0.40 (0%) | 0.49 (0%) | 0.58 (0%) | 0.33 (0%) |
|  |  | **gamma** | -0.34 (0%) | -0.63 (3%) | -0.17 (0%) | 0.02 (0%) |
|  | **Interaction** | **theta** | 0.18 (0%) | 0.50 (2%) | 0.54 (0%) | -0.00 (2%) |
|  |  | **alpha** | 0.32 (0%) | 0.38 (0%) | -0.27 (0%) | 0.29 (0%) |
|  |  | **beta** | -0.49 (0%) | -0.66 (0%) | -0.38 (0%) | -0.08 (0%) |
|  |  | **gamma** | 0.12 (0%) | 0.03 (0%) | 0.29 (0%) | -0.13 (0%) |

***Supplementary Table 6.*** *Coherence between 64-channel human EEG and mouse EEG at 4 electrode sites across frequency bands (theta, alpha, beta, gamma) for each experimental condition. Average effect sizes are reported, with the percentage of significant (after multiple comparison correction) pairwise comparisons in parentheses.*

|  | WT-S | WT-BT | Ca-S | Ca-BT |
| --- | --- | --- | --- | --- |
| BW (g, Prior to the treatment) | 26.43 ± 3.55 | 25.44 ± 3.03 | 25.75 ± 3.21 | 25.03 ± 3.16 |
| BW (g, At termination) | 25.21 ± 3.33 | 24.71 ± 3.74 | 26.06 ± 4.03 | 25.73 ± 3.88 |
| Liver weight (g, at termination) | 1.16 ± 0.05 | 1.09 ± 0.07 | 3.39 ± 1.18 | 2.78 ± 0.97 |

Note: For body weight (BW), the data are presented as Mean ± SD (n=8 per group). For liver weight, data are presented as Mean ± SE (n = 8 per group). WT-S = Wild Type Sham (control); WT-BT = Wild Type Biofield Therapy; Ca-S = Transgenic Cancer Sham; Ca-BT = Transgenic Cancer Biofield Therapy;

***Supplementary Table 7.*** *Body weight (BW) and liver weight across groups.*


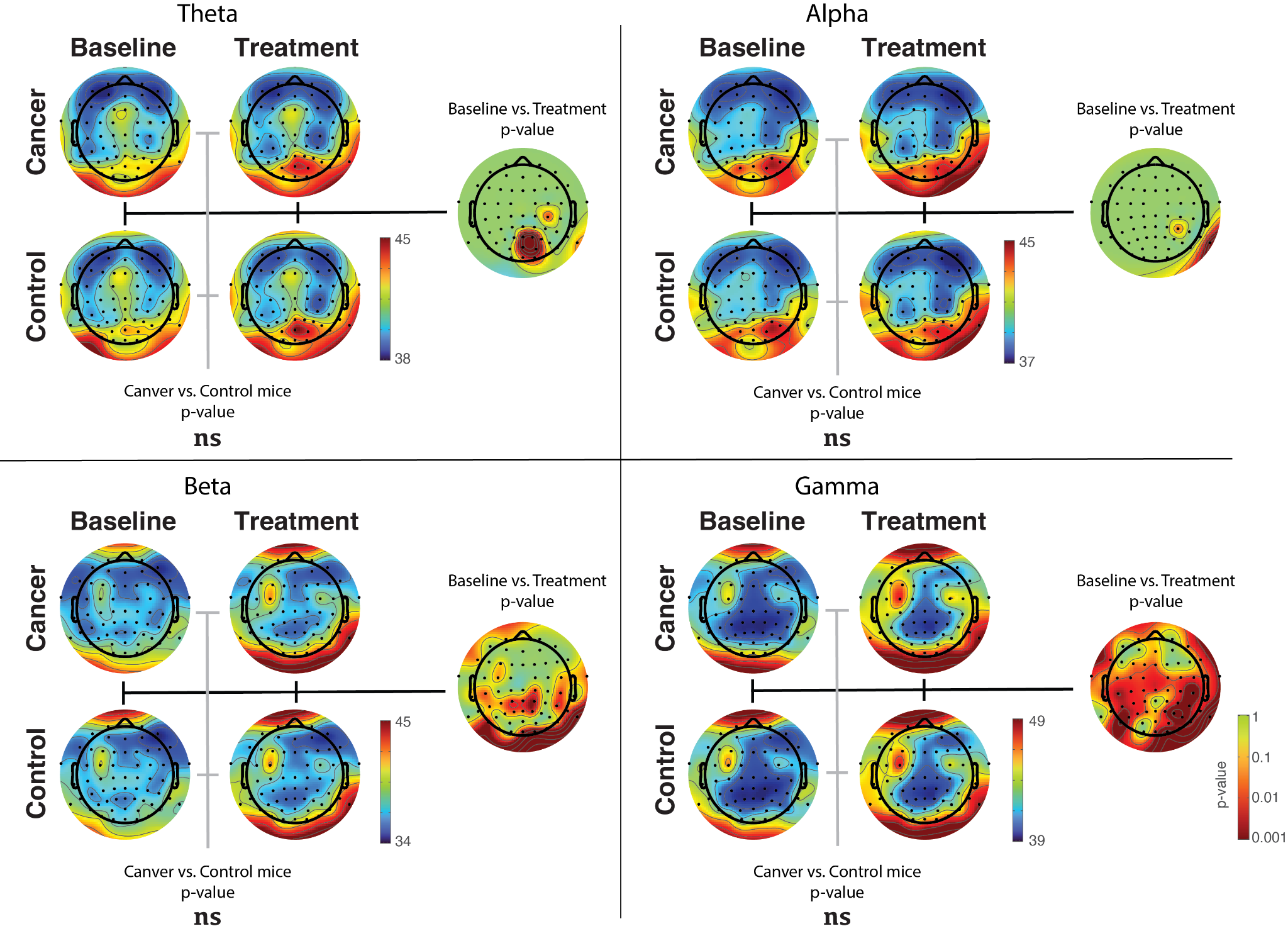


***Supplementary Figure 1.*** *Sham participant EEG spectral power in all frequency bands in the 2 x 2 ANOVA design (mouse type x treatment conditions). Spectral power is shown in the central region of each panel, and significance is shown on the right for the treatment effect and below for the mouse type effect after correction for multiple comparisons. The significance scale (after FDR correction for multiple comparisons) is shown using a logarithmic scale.*

*
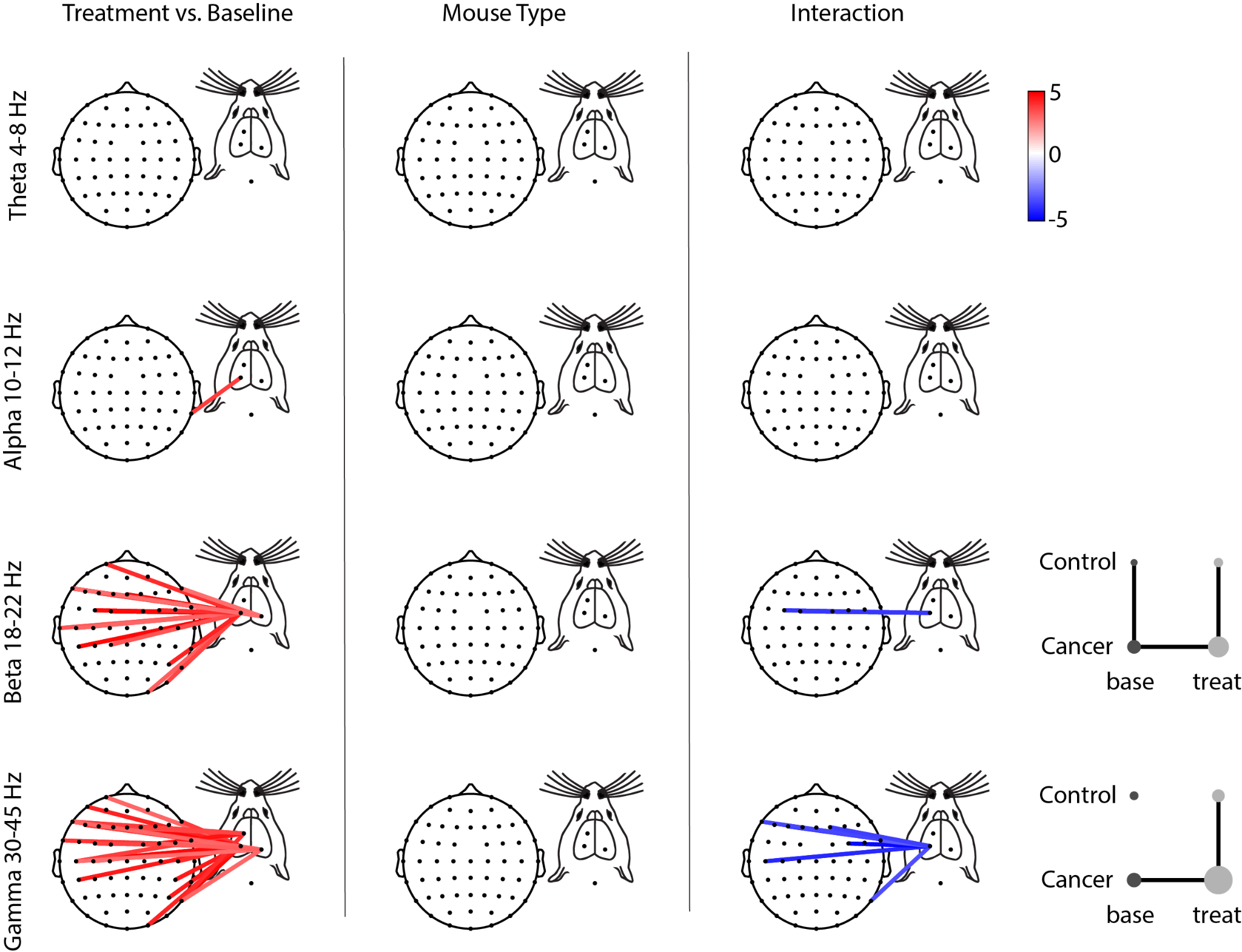
*

***Supplementary Figure 2.*** *Spectral correlation between mouse and sham participant EEG signals across frequency bands. Each row represents a different frequency band, and columns show the effects of treatment vs. baseline (left), mouse type (middle), and their interaction (right). Lines indicate significant correlations between EEG electrodes in mice and humans, color-coded by direction (red: positive, blue: negative). In the interaction column, marginal means for the significant channel pairs are shown as detailed in Figure 4. Statistical significance was determined using mixed models and FDR-corrected (see Methods).*


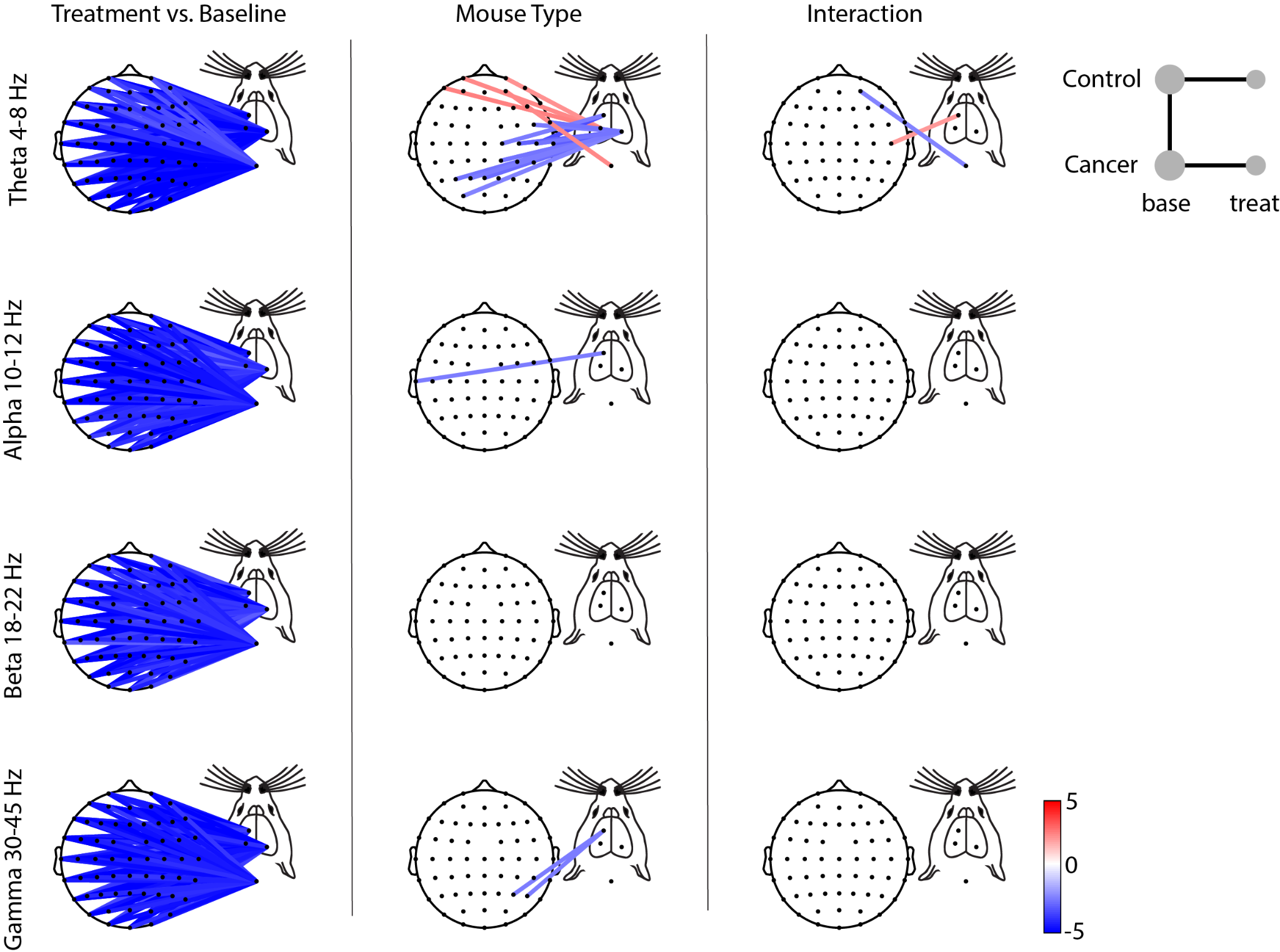


***Supplementary Figure 3.*** *Spectral coherence indicates the synchronization between mice and sham participant's EEG signals across frequency bands. Each row represents a different frequency band, and columns show the effects of treatment vs. baseline (left), mouse type (middle), and their interaction (right). Lines indicate significant correlations between EEG electrodes in mice and human, color-coded by direction (red: positive, blue: negative). In the interaction column, marginal means for the significant channel pairs are shown as detailed in Figure 4. Statistical significance was determined using mixed models and FDR-corrected (see Methods).*
